# Evolution of Neutral and Flowering Genes along Pearl Millet (*Pennisetum glaucum*) Domestication

**DOI:** 10.1371/journal.pone.0036642

**Published:** 2012-05-14

**Authors:** Ghayas Lakis, Miguel Navascués, Samah Rekima, Mathieu Simon, Marie-Stanislas Remigereau, Magalie Leveugle, Najat Takvorian, Françoise Lamy, Frantz Depaulis, Thierry Robert

**Affiliations:** 1 Laboratoire Ecologie Systématique et Evolution, UMR 8079 Université Paris-Sud, Orsay, France; 2 Centre de Biologie pour la Gestion des Populations, Institut National de la Recherche Agronomique Campus International de Baillarguet, Montferrier-sur-Lez, France; 3 Station de Génétique et Amélioration des Plantes, INRA Versailles, Versailles, France; 4 Molecular & Computational Biology, University of Southern California, Los Angeles, California, United States of America; 5 Université Pierre et Marie Curie, Paris, France; 6 UVSQ, Dep Biologie, 45 Bd Des Etats-Unis, Versailles, France; 7 Laboratoire Ecologie et Evolution UMR7625 CNRS-ENS-Université Pierre et Marie Curie 46 rue d’Ulm, Paris, France; North Carolina State University, United States of America

## Abstract

**Background:**

Pearl millet landraces display an important variation in their cycle duration. This diversity contributes to the stability of crop production in the Sahel despite inter-annual rainfall fluctuation. Conservation of phenological diversity is important for the future of pearl millet improvement and sustainable use. Identification of genes contributing to flowering time variation is therefore relevant. In this study we focused on three flowering candidate genes, *PgHd3a*, *PgDwarf8* and *PgPHYC*. We tested for signatures of past selective events within polymorphism patterns of these three genes that could have been associated with pearl millet domestication and/or landraces differentiation. In order to implement ad hoc neutrality tests, a plausible demographic history of pearl millet domestication was inferred through Approximate Bayesian Computation by using eight neutral STS loci.

**Results:**

Domesticated pearl millet exhibited 84% of the nucleotide diversity level found in the wild population. No specific polymorphisms were found either in the wild or in the domestic populations. The Bayesian approach and previous studies suggest that gene flow between wild relatives and domesticated pearl millets is a main factor explaining these results. Early and late landraces did not show significant genetic differentiation at both the neutral and the candidate loci. A positive selection was evidenced in *PgHd3a* and *PgDwarf8* genes of domestic forms but not in the wild population.

**Conclusion:**

Our results strongly suggest that *PgHd3a* and *PgDwarf8* were likely targeted by selection during domestication. However, a potential role of any of the three candidate genes in the phenological differentiation between early and late landraces was not supported by our data. Reasons why these results contrast with previous results that have shown a slight but significant association between *PgPHYC* polymorphisms and variation in flowering time in pearl millet are discussed.

## Introduction

Plant domestication is often considered as a two step evolutionary process. The first step corresponds to the evolution of characteristic traits of domestic phenotypes under the selective pressures that have occurred under the conditions of cultivation of wild populations by the first farmers, as early as the beginning of the domestication [1]. These genetically modified traits define the so-called “domestication syndrome” which is generally shared by all the components of the domestic gene pool of the same species [2], [3].

The second step, concerns the diversification of the domestic gene pool as a byproduct of local adaptations to new environments and to human needs and tastes. This further evolution has produced the morphological and physiological diversity of the domestic gene pool which largely exceeds what is usually observed in their wild counterparts.

Among the traits usually considered as targets of this second step of the domestication process, cycle length has been crucial for domestic populations to cope with new environmental conditions encountered during the expansion of the cultivation areas [4], [5].

As for many other cereals, pearl millet domestication has produced many landraces displaying a strong diversity of their cycle length. This diversity is partly due to photoperiod sensitivity variation among landraces. Pearl millet landraces can be classified according to their cycle length and photoperiod sensitivity. The early varieties (45–70 days between sowing and the beginning of flowering), abundant in the northern dry regions of the Sahel [6], [7], are mostly facultative short-day plants but photoperiod insensitive genotypes have also been reported [8], [9], [10]. On the other hand the semi-late (70–100 days to flowering) and late landraces (100–150 days to flowering), are more abundant in the wetter southern regions. They are considered as strictly sensitive to day length (absolute short-day plants) [8], [9], [10]. Although there is an important variation in flowering time within each group of landraces [9], we have recently shown, in a case study, that the distribution of flowering times among early and late landraces are clearly distinct [11].

Up to date, the history of pearl millet domestication is poorly known [12], [13]. Several scenarios have been proposed on the basis of the geographical patterns of molecular marker polymorphism but no consensus has yet been reached (see a brief review in [13]). In particular, the origin of the diversity in cycle length (i.e. the origin of early and late pearl millet) has been poorly discussed. Tostain et *al.* [14] and Tostain and Marchais [15] suggested that the domestication of pearl millet in West Africa generated essentially early ﬂowering landraces. This step would have been followed at the west side of the actual Lake Chad by a secondary diversification, which would be at the origin of late flowering landraces and new early flowering landraces of East and South Africa and of India.

The diversity in cycle length plays a major role in Sahelian agrosystems where farmers generally grow both early and late (or semi-late) landraces to cope with the uncertainty of the rainy season. This diversity contributes therefore to ensure regularity in grain production [16]. Other reasons are also invoked by farmers to explain their practice of growing these two distinct varietal types, such as the adaptation of early and late landraces to different type of soils and differences in culinary uses. Also, the position of these two varietal types in the cropping calendar and in the management of agricultural lands allows farmers to cope with the seasonal wanderings of transhumant herds (fields grown with early pearl millet are released when transhumant herds arrive from northern regions). The farmers’ knowledge and practices including classification and careful choice of these two varietal types correspond actually to a functional subdivision and a smart biodiversity management tool in the agrosystems. However, it has recently been stated that an evolution to earliness of pearl millet landraces due to adaptation to recurrent droughts would have occurred during the past decades, at least in some parts of the Sahel [6], [17]. The occurrence of gene flow between early and late landraces has been reported despite differences in mean flowering time [11]. This gene flow is driven by recent changes in farmer’s practices due to ecological and social changes in the Sahel of Africa [18]. Furthermore Lakis et *al.* [11] have proposed that, this gene flow and the subsequent introgression could result in a drastic erosion of cycle length diversity in pearl millet. Conservation of this cycle length diversity is of primary importance for the future of pearl millet improvement and sustainable use. The identification of genes contributing in cycle diversity is consequently relevant to this goal.

The genetic and molecular factors underlying the diversity of cycle length in pearl millet are very poorly known since only a few and very recent studies have been devoted to this topic [7], [19]. However in *Arabidopsis thaliana* the flowering pathways and gene networks involved in the floral transition are well described. Four pathways have been shown to be responsible for the control of this process. The autonomous pathway responds to internal signals (such as the developmental stage) independently of environmental signals to initiate flowering [20]. The light dependent pathway is responsible for the perception and integration of changes in light quantity and quality [21]. The Gibberellic Acid (GA) pathway promotes flowering through hormonal signals [22]. The fourth pathway is the vernalization pathway, associated with cold periods to initiate the flowering process [23], which is a characteristic of species living in the temperate zones. A well known feature of the flowering network is that signals from different pathways are integrated to initiate flowering in the shoot apical meristem (SAM) cells by the protein coded by the *FLOWERING LOCUS T* gene [24], traveling from the leaves [25].

It is now well established that grasses and *A. thaliana* share most of the genes involved in flowering time controlling pathways. For example, potential functional orthologous of the *FT Arabidopsis* gene have been identified in rice (*Hd3a*), maize (*ZCN8*), wheat (*VRN-B3/TaFT*) and barley (*VRN-H3/HvFT*) [26], [27], [28]. Potential orthologous of CONSTANS (*CO*), an integrator of the light dependent pathway in *Arabidopsis* [29], have also been identified in those species: *Hd1* (rice), *conz1* (maize), *TaHd1-1*(wheat), and *HvCO1* (barley) [30], [31], [32], [33]. Moreover, it is also now known that grasses have developed original mechanisms involved in the floral transition [34]. For example, an alternative rice specific, light dependent pathway is controlled by a gene with no identified orthologous in *Arabidopsis*, *Ehd1* [35].

In this study we used a candidate gene approach in order to test whether three orthologous in pearl millet of flowering genes already identified in other species have been targeted by selection during domestication. The three genes were *PgHd3a*, *PgDwarf8* and *PgPHYC*. They are respectively orthologous of the *Arabidopsis FT* and the rice *Hd3a*, of the maize *Dwarf8*, and of the *Arabidopsis* and Sorghum *PHYC*.

These three genes have been reported to be involved in cycle length diversity in crops. In rice, the *Hd3a* gene was identified as a QTL which control heading time [36]. Like *FT*, *Hd3a* integrates signals from the different flowering pathways. It has been shown that *Hd3a* haplotypic diversity is associated with flowering time variation of rice cultivars [37]. Similarly, orthologous of *FT* in wheat seem to be involved in the heading date variation observed within a collection of wheat inbred lines from diverse geographical origins [38]. *Dwarf8* was one of the firstly discovered flowering time QTLs in maize [39], [40]. *Dwarf8* is the orthologous of the *Arabidopsis Gibberellin Insensitive* (*GAI*) gene and of the *Rht* gene in wheat [41]. Polymorphisms in the *Dwarf8* gene have been shown to be associated with flowering time variation observed among maize inbred lines [39]. It was also suggested by Camus-Kulandaivelu *et al.* [42] that *Dwarf8* may have been involved in the adaptation of maize to new latitudes through diversifying selection on the heading date trait. The *PHYC* gene encodes for a photoreceptor protein. It was related to flowering time variation and local adaptation among *Arabidopsis* accessions [43]. *PHYC* has also been shown to contribute to variation for cycle length in pearl millet. Indeed Saïdou *et al.* [19] found a weak but significant association between polymorphisms in the *PgPHYC* gene and flowering time variation among a collection of several inbred lines originated from India, West and East Africa.

In this study we first cloned the coding sequences of *PgHd3a* and *PgDwarf8*. We also cloned a region of the *PgPHYC* gene which corresponds to the third intron and part of the fourth exon of the maize *PHYC* gene. Secondly, we evaluated the level of genetic differentiation between wild and domestic pearl millets, and between early and late landraces on the basis of nucleotide polymorphism in a collection of domestic landraces (early and late) and wild accessions for both candidate genes and control loci. These domestic and wild pearl millets originated from the whole geographical distribution area of pearl millet in the Sahel of Africa. Finally, we tested whether nucleotide polymorphism pattern at these three candidate genes is consistent with a recent selection event and a potential role in the differentiation of early and late landraces. It is well known that the effects of selection and demographic history on nucleotide polymorphisms patterns are difficult to disentangle [44]. To circumvent this problem we implemented ad hoc neutrality tests. We used polymorphism data at eight presumably neutral loci to infer the demographic parameters of a plausible history of the pearl millet domestic populations. Approximate Bayesian Computation approach [45] and coalescent simulations were used to generate the expected distribution of neutrality tests that fitted the observed data better than the strict Wright-Fisher neutrality model.

## Methods

### Molecular Polymorphism Analyses: PCR, Cloning and Sequencing

The primers used for this study are listed in Table S1. Primers used for the candidate gene *PgHd3a* were designed using the rice *Hd3a* and the *Arabidopsis FT* orthologous sequences (respectively [BD169090.1] and [AB027504]). The primers used for the *PgDwarf8* gene were designed on the basis of its orthologous sequence in maize [AF413202.1]. Primers of the *PgPHYC* gene were designed from the *PgPHYC* fragment isolated by Saïdou *et al.* [19].

The three candidate genes and eight STS single copy loci (≈9800 bp), were PCR amplified using the Invitrogen “Platinum Taq DNA Polymerase High Fidelity” on a collection of wild and domestic pearl millet accessions (in average 22 early and 20 late) originated from the whole geographic distribution area of this species in Africa (Table S2). Six accessions from Asia were also included (Table S2). Three of these eight STS loci were previously sequenced on a subset of accessions [46].

Each locus was amplified and sequenced from one individual per accession. In some cases the STS loci and the candidate genes could not be amplified in a given individual for technical reasons. Therefore, PCR amplification was done on another individual from the same population, or, when this was not possible, on another accession corresponding to a geographically close population (Table S2). The PCR products of the candidate genes and of the STS 738 were cloned and sequenced using the TOPO-TA (Invitrogen). For each of the other STS locus, PCR products from at least 10 individuals were cloned and sequenced in order to obtain the true haplotypes (see below). These latter were used as references to infer the most likely haplotypes from other sequences obtained from direct sequencing of the PCR products. PCR products were sequenced in forward and reverse directions in order to exclude sequencing errors. To test for Taq polymerase errors in the sequence data, PCR amplification and sequencing were carried out two times for 10 individuals and for each of the *PgHd3a* gene and the STS 713 loci. No errors were found on the 20 re-sequenced fragments (19830 bp). In addition the STS 306 showed only one SNP out of 38248 sequenced bp. If this SNP was a Taq polymerase error, this result would imply an error rate of <3 errors for every 10^5^ bp**.** This value is congruent with estimations of the error rate (2×10^−5^ errors per bp) for the High Fidelity (Invitrogen) Taq DNA Polymerase [47]. All sequences were deposited in Genbank under accession numbers [JQ001940-JQ002518].

### Isolation of the PgHd3a cDNA

23DB inbred line plants were grown in 16 h day and 8 h night (long day conditions) during one month. Plants were transferred in short day (SD) conditions: 12 h day and night, to induce floral transition. Total RNA was extracted from leaves collected in the morning from plants grown for 2 weeks in SD conditions using Trizol (Invitrogen). The *PgHd3a* forward primer was used to perform the 3′ Rapid Amplification of cDNA Ends (RACE) following recommendations for the RACE kit (Clonetech). cDNA was synthesized using Superscript II reverse transcriptase (Invitrogen). The amplification products were cloned in TOPO-TA vector (Invitrogen) in order to obtain the sequence of the *PgHd3a* full transcript.

### Statistical Analyses of Molecular Polymorphisms

Base calling, quality assessment, and sequence assembly were conducted using CodonCode Aligner (V.3.5.7). ClustalW implemented in the Bioedit [48] software was used to perform the multiple sequences alignment of each locus. Accessions that were undetermined for their cycle length (see table S2) were not included in analyses conducted on early and late accessions separately. Polymorphism and molecular evolution analyses were performed using DnaSP v5.00 [49] and Fabsim software [50].

#### Haplotype inference

In order to infer the allelic phase of the STS sequences obtained from direct sequencing of PCR products, the algorithms PHASE v2.1 [51], [52] implemented in DnaSP [49] was used. One of the two inferred haplotypes was randomly chosen for further statistical analyses.

#### Assessment of the genetic structure within the collection of accessions

The Arlequin software (version 3.5) [53] was used to estimate *F*-statistics. The significance of each pairwise *F*st value was assessed by performing 10000 permutations. The SNP data generated from the concatenated STS fragments was used to evaluate the genetic structure within the collection of accessions by using the Bayesian method implemented in the Structure software version 2.3.3 [54]. 500,000 iterations were carried out for each run after a burn-in period of 100,000 iterations. The model allowing for admixture and correlated allele frequencies between populations was used for this analysis. Five independent replicates for each value of K (the number of a priori clusters) were performed. This procedure was repeated for K varying from 1 to 10. The optimal number of clusters (K) was identified by using the *ad hoc* statistics based on the second order change of the likelihood function with respect to K (ΔK) developed by Evanno *et al.* [55].

### Bayesian Inference of the Demographic History of Pearl Millet Domestic Population

A plausible demographic history of domestication was inferred using an approximate Bayesian computation (ABC) analysis [45] on the basis of summary statistics estimated from the molecular polymorphism data at the eight STS loci. Many simulated data sets were generated from the model with parameter values taken from prior distributions. The posterior probabilities distributions were estimated on the basis of the most likely simulated data. The latter was identified through estimation of distances between summary statistics of the simulated data and the real data set (see [45] for a review on ABC).

The demographic model simulated in this study consisted of a constant sized wild population from which an exponentially expanding (domestic) population originates at domestication time. Two versions of this model were used. In the first one, gene flow occurs between wild and domestic populations. The second version did not include the occurrence of gene flow. The parameters of this model were: the scaled (i.e. scaled to coalescent units) mutation rate for the wild population (per bp, θ_W_ = 4Ne_W_µ); the scaled mutation rate for the domestic population at present (θ_D1_ = 4Ne_1_µ) and at the domestication time (θ_D0_ = 4Ne_0_µ); the scaled time of domestication (T = t/4Ne_W_); the scaled recombination rate (for consecutive bp, ρ = 4Ne_W_r. ); the scaled migration rates for gene flow in both directions: migration from wild to domestic (4Nem_W→D_) and migration from domestic to wild (4Nem_D→W_). µ is the mutation rate per generation per bp, t is the time of domestication in generations unit (1 year per generation for annual plants as pearl millet), and r is the recombination rate (for consecutive bp per generation). It must be noticed that eventual variations of the neutral mutation rate and of the recombination rate among loci are neglected in this model.

Uninformative prior probability distributions were used for θ_W_, log-uniform (min = 10^−3^, max = 1), θ_D1_, log-uniform (min = 10^−8^, max = 1), ρ, log-uniform (min = 10^−50^, max = 1); and the migration rate, uniform (min = 0, max = 100). On the basis of archeological records, pearl millet domestication is believed to have been achieved between 3,000 to 4500 years ago [56], [57], [58]. However, it is very likely that agrarian societies on the African continent had a Neolithic way of life since at least the eighth millennium BC [59], [60]. In consequence, a uniform prior was set (min = 3000; max = 12000) for the parameter t (time of domestication in generations unit). The prior for θ_D0_ was set to be conditional to the values of θ_D1_ and θ_W_ so that it always took a value lower to both of them. This was done to ensure the simulated domestication scenario includes a demographic bottleneck with respect to the wild population immediately followed by a demographic expansion. The prior distribution for θ_D0_ was log-uniform with maximum set to the lowest value between θ_D1_ and θ_W_ and the minimum set to 10^−5^ times that value. In order to scale domestication time in generations to coalescent time (4Ne_W_ generations unit), the mutation rate was also considered. Mutation rates for maize are estimated to be between 3×10^−9^ and 1.5×10^−7^ per bp per year (with point estimates around 3×10^−8^) [61]. Thus a log-normal prior (log-mean = −17.7, log-s.d. = 1) on µ was chosen in order to cover the range of these estimated values and their uncertainty. Simulations (10^6^ for each of the two scenarios and each locus) were performed with ms [62]. Each locus was simulated separately to take into account the differences in sample sizes and in the length of these sequences.

Intra-population and inter-population summary statistics were computed for real and simulated data in order to compute a distance measure between real and simulated data for the ABC analysis. The best fitting model was chosen based on the posterior probabilities for the two demographic scenarios (absence or presence of gene flow between wild and domestic millet). These posterior probabilities were estimated by the logistic regression approach [63] from the closest 2×10^5^ simulations from both scenarios. For the chosen model, posterior probability density distributions for the parameter values were estimated by the non-linear regression ABC approach [64] from the closest 2×10^5^ simulations. Point estimates (median) and 95% highest posterior density (HPD) intervals were estimated from these posterior distributions.

Single-population summary statistics used to describe diversity in both the wild and the domestic populations were mean and variance among loci for: expected heterozygosity (He), nucleotide diversity (π), number of exclusive polymorphisms, variance of pairwise differences and Tajima’s D [65]. Because the domestic population undergoes a more complex demographic history, additional summary statistics were computed on that population: mean and variance among loci for the frequency of the most common haplotype, number of singletons and raggedness index [66]. Two-population statistics included the mean and variance across loci for: total expected heterozygosity, minimal pairwise genetic distance between populations, mean pairwise genetic distance between populations, variance of pairwise genetic distance between populations, number of fixed differences and number of exclusive and shared polymorphisms [67]. In addition, three statistics (RS, Wx2s1 and Wx1s2, unpublished data of Navascués et al.), based on the spatial distribution along the nucleotide sequence of fixed differences and exclusive and shared polymorphism between the wild and the domestic population were used. These statistics are sensitive to the presence and direction of gene flow. For these statistics, the mean across loci and the Kolmogorov-Smirnov D statistic of the comparison between the distribution of the normalized statistics among loci and the standard normal distribution were used. The behavior of these statistics in respect to differentiation and gene flow will be published elsewhere.

Sampling parameter values from prior probability distributions, running ms, calculation of summary statistics and estimation of posterior probabilities were performed in R (R Development Core Team 2009); a script is available from M. Navascués on request. Posterior probabilities were estimated with the R package “abc” (http://cran.r-project.org/web/packages/abc/index.html).

### Tests for Selective Neutrality of Candidate Genes

Genetic diversity in candidate genes (*PgHd3a*, *PgDwarf8* and *PgPHYC*) were tested against the neutral hypothesis including the demographic history of domestic populations as inferred from the ABC analysis. Null distributions of summary statistics were estimated by coalescent simulations (10^4^) using parameters values randomly drawn from the joint posterior probability distributions. The number of polymorphic sites (S) in each simulation was fixed to the observed value. Tajima’s D [65] and Fu & Li F* [68] statistics and their associated *p*-values were calculated for each pseudo-sample. Simulations were performed with ms [62] and summary statistics were calculated from the ms output files with Fabsim [50].

## Results

### Nucleotide Diversity and Polymorphism within the STS Fragments

#### Identification of sequences similar to the Sequenced Tagged Sites in grasses

A search of Genbank using BLASTn indicated that the STS 344, the STS 359 and the STS 521 have no similarities with other known nucleotides sequences. The STS 870 shared similarity with an mRNA of a hypothetical protein of sorghum (e-values 4e-42) and maize (e-values 2e-40). The STS 306 shared also similarities with a hypothetical sorghum protein (e-value 9e-68). Remigereau et *al.* [46] showed that the STS 713 shared high similarity with multiple plant protein kinases from the RLG family in maize, sorghum and rice (e-values 3e-69 to 4e-04) and that the STS 476 shared high similarity with an mRNA of a hypothetical protein in Sorghum (e-value 3e-165). They also revealed that the STS 738 showed similarity with a single rice BAC clone (e-value 6e-07).

#### Pattern of STS polymorphism within the domestic and wild populations

In comparison with other outcrossing wild relatives of crops, wild pearl millet showed a lower level of nucleotide diversity. Indeed, the nucleotide diversity (π) of the wild pearl millet was 0.0062 on average whereas it is 0.0095 in teosinte [69] and 0.012 in *H. annuus* [70] who are also outcrossing species. However it is higher than the nucleotide diversity of the highly selfing *Glycine soja* (0.0022) [71] and *Triticum turgidum* ssp. *dicoccoides* (0.0027) [72]. Interpretation of this result is not straightforward, in particular because the level of neutral genetic diversity is not only influenced by the mating system, but also by the demographic history of populations, which is largely unknown for wild relatives of crops. For example, the various climatic periods during the last 20000 years (the maximal age of the last glacial era) in Africa [73], including the recent drought episodes, could have strongly modified the distribution and the demography of grasses, among which the wild *Pennisetum glaucum*, and therefore their genetic diversity.

Our results also showed that the nucleotide diversity of the domesticated pearl millet is relatively high compared to other major crops. Indeed the nucleotide diversity of the domestic samples estimated by π ranges from 0.0021 to 0.0101 (depending on the STS locus considered) and its average value was 0.0054. This last value is very close to the maize nucleotide diversity (0.0063) [69] and almost identical to the nucleotide diversity of barley (0.0051) [74], but higher than the nucleotide diversity found in sorghum (0.0022) [75] and rice (0.0023) [76]. However, these estimations were obtained on non homologous loci. This hinders comparison of the level of nucleotide diversity among species since it is possible that the evolutionary rates of the loci studied in the different species are very different due to differences in the evolutionary constraints suffered by these non homologous loci. This is especially true for pearl millet because it is not known whether the STS loci are coding sequences.

The pearl millet average ratio π_domestic_/π_wild_ for the STS loci (Table 1) showed that the domestic populations of pearl millet are 16% less polymorphic in average than the wild populations. This was expected since plant domestication is generally associated with a loss of diversity due to the contribution of only a subset of the wild populations to the domestic gene pool. It is noticeable that no specific sites and haplotypes were found either in the wild or in the domestic populations. As far as we know, the loss of diversity we observed on the domesticated pearl millet is one of the smallest among all studied cereals [77].

**Table 1:** Genetic diversity and tests for strict neutrality for the eight STS loci.

		Sample size	Length	S^a^	Sin^b^	Specific sites^c^	π^d^ (10^−3^)	θ^d^ (10^−3^)	LSin^e^	Dom/Wild π ratio	Tajima’s D^f^	L group^g^
STS 306	Dom.	51	563	5	2	1	2.13	1.97	2	0.72	0.20 ns	4
	Wild	17	563	5	1	1	2.95	2.63	1		0.40 ns	
STS 344	Dom.	43	710	33	15	20	10.1	11	12	1.06	−0.27 ns	4
	Wild	12	710	20	6	6	9.5	9.5	2		−0.02 ns	
STS 359	Dom.	49	440	15	6	8	7.43	8.86	6	0.78	−0.20 ns	2
	Wild	18	440	15	6	6	9.53	10.72	4		−0.46 ns	
STS 476	Dom.	61	752	10	5	6	2.09	2.86	5	0.52	−0.74 ns	3
	Wild	15	752	14	8	9	4.03	5.92	4		−1.26 ns	
STS 521	Dom.	46	617	29	16	16	5.34	11.56	11	0.86	−1.80*	5
	Wild	19	617	25	21	10	6.18	12.35	10		−1.95*	
STS 713	Dom.	51	1065	30	18	20	2.87	6.62	12	0.44	−1.87*	6
	Wild	22	1065	32	16	19	6.45	8.25	9		−0.83 ns	
STS 738	Dom.	29	1995	64	47	48	4.93	8.32	21	0.92	−1.54 ns	2
	Wild	12	1995	42	26	24	5.33	7.07	10		−1.12 ns	
STS 870	Dom.	49	744	36	18	28	8	12.87	13	1.4	−1.19 ns	6
	Wild	20	744	23	14	9	5.78	8.94	7		−1.36 ns	
Average	Dom.	47	860	28	16	18	5.36	8	10	0.84	−0.93	
	Wild	17	860	22	12	11	6.22	8.17	6		−0.83	

a S: number of segregating sites. ^b^Sin:number of singletons. ^c^Specific sites: sites polymorphic in one population but monomorphic in the other. ^d^θ: Watterson estimator of sequence diversity per site, π: average number of differences per site between two sequences. ^e^Number of lineages with at least one singleton. ^f^D values were calculated using Fabsim [50], *P-*values of D were calculated for the standard coalescent model (*: p<0.05; ns: not significant); *P*-values were computed without correction for multiple tests.^ g^linkage group of the pearl millet genetic map on which the locus is located.

Our data also showed that the polymorphism revealed by the STS loci can be considered as generally neutral in both the wild and in the domestic populations when tested against the standard Wright-Fisher neutral model. The wild and the domestic pearl millet showed a slight excess of rare polymorphism as shown by negative values of Tajima’s D. This result was unchanged when the six Asian accessions were removed (data not shown). The singletons were distributed on a high number of lineages suggesting that the STS loci for both the domestic and wild populations had a star shaped genealogy (Table 1). However, Tajima’s D values were only slightly significant for the STS 713 in the domestic populations and the STS 521 in the wild and domestic populations (Table 1).

#### Comparisons of nucleotide diversity between early and late landraces on the basis of STS locus

The early and the late landraces revealed almost the same nucleotide diversity level (Table S3). In fact, the average values of π for the early landraces and for the late landraces were almost identical (0.0055 and 0.0051, respectively). Finally, neutrality tests implemented separately on early and late pearl millet landraces gave similar results in the two groups of plants (Table S3).

Very few studies have compared the genetic diversity of different phenological groups in cereals. Results similar to ours were obtained for maize inbred lines on the basis of 1095 sequenced genes. The early temperate populations and the late tropical populations were found to have almost equal values of nucleotide diversity (0.0065 and 0.0061) [78].

### Genetic differentiation between Wild, Early and Late Pearl Millets and Levels of Genome Admixture

**Figure 1:**
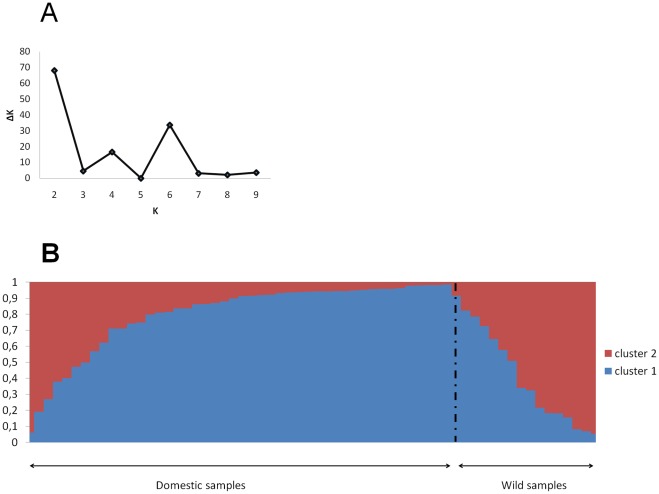
Genetic structure of wild and domesticated populations. A. Values of ΔK calculated by using the Evanno et al. (2005) method according to the number K of clusters B. Representation of the individual assignment probabilities to each of the 2 inferred clusters; individuals were arranged according to the estimated proportion of admixture in their genome.

Our results showed that the wild and the domestic populations are significantly genetically differentiated, as suggested by *F*st values (Table S4) and by the analysis of the genetic structure within the collection of accessions. The average *F*st value between the wild and the domestic populations across all STS loci was 0.12. All the *F*st values estimated on each STS locus were significant, except for STS 521 and STS 738. The Bayesian analysis of the population genetic structure showed that the most likely number of groups was K = 2 (Figure 1A). Figure 1B shows that each of the two clusters was composed of both domestic and wild individuals. The wild pearl millets showed high level of genome admixture from both clusters. Genome admixture was also found for the domestic individuals, but in a lesser extent. This pattern may be due to either shared ancestral polymorphisms or migration between the two forms of pearl millet. The difference in genome admixture proportion between wild and domestic individuals suggested that the gene flow may be asymmetrical with preferential introgression of wild genotypes by genes from the domestic populations.

The STRUCTURE analysis dedicated to the domestic populations only did not reveal any clear clustering of the individuals according to either their phenological type or their geographic origin (data not shown). In addition, *F*st values (Table S4) between early and late accessions were not significant across all loci (average *F*st = 0.04) except for STS 306 and STS 344. Altogether, these results confirmed that the differentiation between the two groups of landraces was weak.

### Bayesian Inference of the Demographic History of Pearl Millet Domestication

The demographic history of pearl millet domestication is largely unknown. As stated above, several scenarios could explain the pattern of genetic diversity observed within the domestic gene pool and its level of genetic differentiation with the wild *Pennisetum glaucum.* In order to get a better insight of what could have been a plausible history of pearl millet domestication, an Approximate Bayesian approach was developed using the supposedly neutral STS loci. This has also allowed us to distinguish, in a second step, the effect of demography from the effect of directional selection on the polymorphism pattern within the three flowering candidate genes. Indeed modifications of polymorphisms pattern in the genome of domestic plants relatively to their wild ancestor have been driven by both the demographic history of domestic population, which is known to affect the whole genome, as well as by positive selection for adaptive mutations, which should affect only domestication genes and surrounding genome regions.

**Figure 2:**
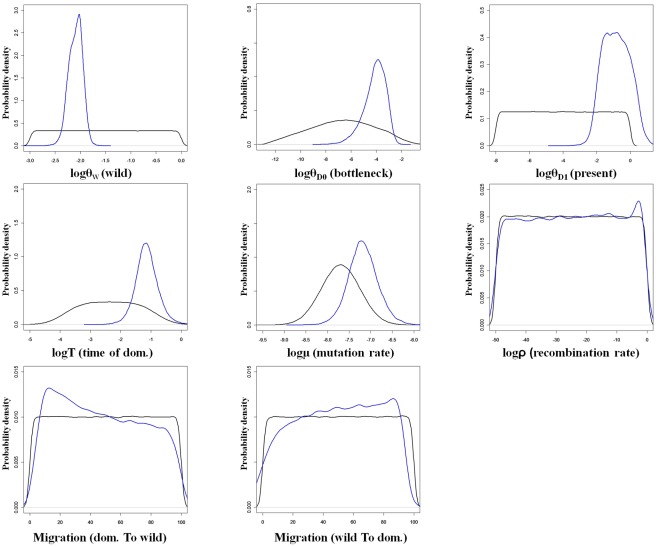
Posterior probability density distribution of the parameters of the demographic model inferred by ABC estimation. Data shown was obtained for the scenario “with migration”. The prior (black line) is used as a reference for the posterior distribution (blue lines) obtained from the rejection followed by the non linear regression. Parameters T, M and rho are scaled to the wild population effective size Ne_w_.

The posterior density probability distributions, of the parameters included in the model, were different from the prior distributions with clearly identified modes (Figure 2). This allowed point estimations of these parameters except for rho, the effective migration rates (Figure 2) and t, the time of domestication in generation units (not shown). Indeed, the posterior distributions of these parameters were very similar to their prior distributions. Thus, our data was not informative enough to get reliable estimates of these parameters.

The “with migration” scenario was only two times more likely (p = 0.637) than the “without migration” scenario (p = 0.363). Neither of the two scenarios could therefore be excluded. Moreover, the estimations of the different parameters from the two models were very close from each other and their confidence intervals broadly overlapped (Table 2, and Table S5). In addition the current occurrence of gene flow between wild and domestic gene pools has been shown in several studies [79], [80], a condition that likely existed since the beginning of domestication. Therefore, only results obtained from the “with migration” scenario are extensively shown and discussed hereafter (Table 2 and Figure 2).

**Table 2:** Estimates of demographic and genetic population parameters obtained from the demographic model “with migration.”

Parameter^a^	median	95% HPD interval^b^
θ_W_	0.0082	0.0042–0.0142
θ_D0_	9.79×10^−5^	6.05×10^−7^–0.0017
θ_D1_	0.1409	0.0060–4.5243
T	0.0704	0.0133–0.4524
µ	6.50×10^−8^	1.39×10^−8^–3.41×10^−7^

a θ_W_ is the population mutation rate of the wild population, θ_D0_ is the population mutation rate of the domestic population at domestication time, θ_D1_ is the population mutation rate of the domestic population at present time, T is the time of domestication in units of 4Ne_W_ generations, and µ is the mutation rate per bp per generation. ^b^HPD interval is the interval of parameter values with the highest posterior density.

The inferences revealed that the estimation of the neutral mutation rate was 6.5×10^−8^/bp/year which is about ten times higher than the only estimation available up to date for a nucleotide sequence (the Adh1 gene) in pearl millet [81].

Our results showed that the posterior distribution for the beginning of domestication “t” in years is very similar to the prior distribution. This is not surprising pertaining to the very narrow time window we have defined for the prior. No direct inference about this parameter can therefore be given. Nevertheless, the posterior distribution of “T” can be used to infer a domestication time in years (one generation per year) by using its median and the medians of the posterior distributions for both the mutation rate (µ) and the wild population mutation rate (θ_W_). We estimated, the time of domestication in 4Ne_W_ generations, as equal to 0.0704 and therefore a domestication time equal to about 8900 years. This makes sense relatively to what archeological data has suggested about the beginning of agriculture in Sahel. We would like to point that this method of calculus does not take into account the uncertainty of the three estimators. Thus, the estimation of T expressed in years should be taken with a lot of caution.

The simulation data showed that the bottleneck severity at the domestication time might have been strong, the estimation of the θ_D0_/θ_W_ ratio being equal to 1.1×10^−2^. However this conclusion should be taken with cautious because of the large 95% credibility interval of this parameter (7.51×10^−5^−0.25). The upper bound of this interval means that the diversity of the founder domestic population at the domestication time could represent up to 25% of the genetic diversity of the wild populations.

We estimated the magnitude of the population expansion after the initial bottleneck by the ratio between θ_D1_ and θ_D0_, the population mutation rates of the current and the initial domestic populations respectively. It was equal to 1.83×10^3^ (with a 95% credibility interval of (12.69−6.56×10^5^)). Again, the large credibility interval on this parameter does not allow a precise estimation of the strength of this expansion. Demographic events undergone by domestic pearl millet populations, such as bottlenecks and expansion, could also explain the high variance of the π_domestic_/π_wild_ ratio among the STS locus that was observed (Table 1).

### Cloning and Characterization of the *FT*(*Hd3a*), *PHYC* and *Dwarf8* Orthologous Loci in Pearl Millet

*PgHd3a.*

The identification of the true orthologous of *Hd3a* and *FT* in pearl millet was not trivial. Indeed, Hd3a (rice), FT and TFL1 (arabidopsis) and their homologous in other species belong to the PEBP (phosphatidylethanolamine-binding protein) super family, a multi-gene highly conserved protein family found in many Eukaryotes species [82]. In the plant model *Arabidopsis*, some of the PEBP family members could act in redundancy to induce flowering such as FT and MFT [83], while other members may act antagonistically, such as FT and TFL1 [84]. Two amino acid residues are thought to be the most critical for distinguishing FT and TFL1 activity in *Arabidopsis*, the Tyr85/His88 and the Gln140/Asp144 (FT/TFL1) [84]. It has been also shown for *Arabidopsis* that the Tyr(Y85)/His(H88) determines whether the protein act as an activator or a repressor of flowering [85].

**Figure 3:**
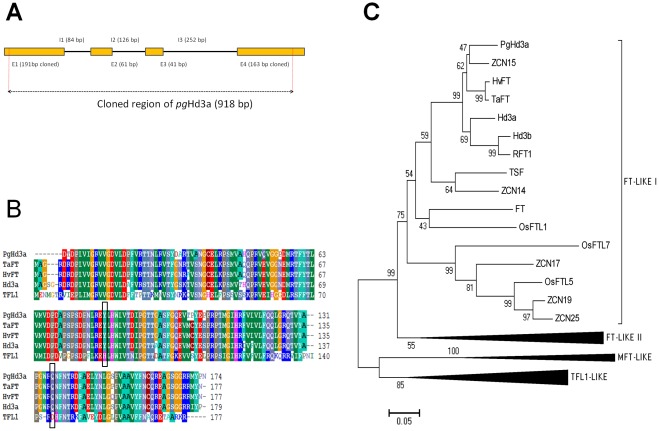
Structure of the pearl millet Hd3a orthologous and sequence similarity with PEBP family members. A. structure of the *PgHd3a* gene, E:exon and I: intron; the 918 bp fragment used in the population genetics analysis is shown between the two dotted red lines; the cDNA sequence was used to complete the 3′ region of the original sequence and to obtain the protein sequence. B. Multiple sequence alignment of the proteins of PgHd3a, the orthologous of FT (wheat (TaFT), barley (HvFT) and rice (Hd3a)) and TFL1; in the black boxes: the Tyr/His(88) and the Gln/Asp(145) residues which are essential to distinguish FT and TFL1. C. A neighborhood joining tree of the PEBP protein family in pearl millet (PgHd3a), maize (ZCN), rice (Os, RFT1, Hd3a, Hd3b), wheat (Ta), barley (Hv) and Arabidopsis (FT). The tree was built with the Poisson corrective distance [96] model. Robustness of nodes was tested on the basis of 1000 Bootstrap iterations.

We first isolated a fragment of 918 bp from PCR amplification showing only one band, using primers designed for Hd3a coding regions. We then sequenced the amplicon and determined the structure of this gene fragment by isolating the cDNA (Figure 3A). We believe that the cloned fragment corresponds to the true orthologous of *Hd3a* in pearl millet (*PgHd3a*) for several reasons:

1. The high similarity of the PgHd3a putative protein with putative proteins in cereals which have been proved to have the same physiological role in promoting flowering, as FT in *Arabidopsis*: 91% of protein sequence identity with TaFT/VRN-B3, 90% with HvFT/VRN-H3 and 87% with Hd3a (Figure 3B).
2. The putative protein of PgHd3a showed in every accession the two amino acids residues that are thought to be the most critical for the flowering promotion activity of FT in *Arabidopsis* (Figure 3B).
3. The sequence of the putative protein encoded by the pearl millet *Pg*Hd3a was in the same monophyletic group as TaFT/VRN*-*B3, HvFT/VRN-H3, Hd3a and FT (Figure 3C).

*PgDwarf8.*

The *PgDwarf8* cloned fragment was 1233 bp. Compared to the maize *Dwarf8*, the amplified fragment in pearl millet had no intron as the maize gene [39]. It started 360 bp from the ATG and finished 148 bp before the end of the exon partial sequence published by Thornsberry *et al.* [39]. The putative protein sequence revealed a high degree of similarity with the maize Dwarf8 protein (93% of amino acids identity).

*PgPHYC.*

The *PgPHYC* fragment cloned in this study was 823 bp, only 43 bp shorter than the sequence amplified by Saïdou *et al*. [19].

The structure of *PgPHYC* is unknown. However the sorghum *PHYC* gene was proven to have 4 exons and 3 introns. The cloned fragment of *PgPHYC* included the whole of the third intron and the first 123 bp of the 4^th^ exon (89% of nucleotide identity with the 4^th^ exon of *Sorghum propinquum*).

### Polymorphism Analysis of the Candidate Genes

*PgHd3a* and *PgDwarf8* showed significant differentiation between the domestic and the wild populations (*F*st = 0.12; and 0.095 respectively) (Table S4) while *PgPHYC* did not reveal such a differentiation (Table S4). Also, within the domestic population, the loss of genetic diversity relatively to the wild population was particularly higher for *PgDwarf8* than for the STS loci (Table 1, Table 3 and Table 4). On the contrary *PgPHYC* showed a gain of diversity in the domestic population in comparison with the wild population.

**Table 3:** Genetic diversity and selective neutrality tests for *PgHd3a*.

		Sample	Length	S^a^	Sin^b^	π^c^ (10^−3^)	θ^c^ (10^−3^)	LSin^d^	Dom/Wild	Tajima’s D^e^	Fu & Li’s F*^e^
		size							π ratio	*P*-values^f^	*P*-values^f^
*PgHd3a*	Dom.	59	918	47	42	2.48	11.2	23	0.72	−2.62**	−5.92**
				nc:24		nc:3.08	nc:11.43				
				syn:8		syn:3.01	syn:15.41				
				nsyn:14		nsyn:1.43	nsyn:9.07				
	Early	26	918	22	19	2.4	6.38	9		−2.26*	−3.69*
				nc:10		nc:2.59	nc:5.8				
				syn:5		syn:4.07	syn:11.72				
				nsyn:7		nsyn:1.62	nsyn:5.52				
	Late	28	918	25	22	2.59	7.07	14		−2.3*	−4.12*
				nc:15		nc:3.6	nc:8.47				
				syn:3		syn:1.87	syn:6.74				
				nsyn:6		nsyn:1.27	nsyn:4.55				
	Wild	14	918	17	11	3.47	5.88	7		−1.69 ns	−1.69 ns
				nc:14		nc:5.99	nc:9.68				
				syn:0		syn:0	syn:0				
				nsyn:3		nsyn:1.27	nsyn:2.79				

a S: number of segregating sites. ^b^Sin: number of singletons. ^c^θ: Watterson estimator of sequence diversity per site, π: average number of differences per site between two sequences, the diversity of the candidate genes was estimated for the non-coding (nc), synonymous (syn) and non-synonymous (nsyn) sites. ^d^Number of lineages with at least one singleton. ^e^D and F* were calculated for the simulation using parameters randomly drawn from the posterior distribution.^ f^ *P-*values were computed for unilateral tests as *P*< or > than the observed value according to the sign of the statistics without correction for multiple tests.

**Table 4:** Genetic diversity and tests for selective neutrality for the two candidate genes: *PgDwarf8* and *PgPHYC*.

		Sample	Length	S^a^	Sin^b^	π^c^ (10^−3^)	θ^c^ (10^−3^)	LSin^d^	Dom/Wild	Tajima’s D^e^	Fu & Li’s F*^e^
		size							π ratio	*P*-values^f^	*P*-values^f^
*PgDwarf8*	Dom.	33	1233	46	40	3.15	9.73	15	0.28	−2.47*	−4.48*
				syn:19		syn:4.97	syn:16.23				
				nsyn:23		nsyn:1.98	nsyn:7.19				
	Early	14	1233	14	11	2.4	3.7	7		−1.39 ns	−2.08 ns
				syn:2		syn:1.9	syn:2				
				nsyn:11		nsyn:2.24	nsyn:4.15				
	Late	16	1233	32	29	3.81	8.02	7		−2.18*	−3.17*
				syn:15		syn:6.69	syn:14.59				
				nsyn:15		nsyn:2.67	nsyn:5.35				
	Wild	12	1233	56	26	11.22	15.26	10		−1.22 ns	−0.76 ns
				syn:32		syn:24.74	syn:32.64				
				nsyn:24		nsyn:6.38	nsyn:9.05				
*PgPHYC*	Dom.	38	823	24	13	5.67	6.96	6	1.45	−0.63 ns	−1.89 ns
	Early	18	823	20	10	6.39	7.07	4		−0.37 ns	−0.93 ns
	Late	17	823	10	2	4.78	3.6	1		1.2 ns	0.81 ns
	Wild	15	823	13	6	3.91	4.86	3		−0.77 ns	−0.73 ns

a S: number of segregating sites. ^b^Sin: number of singletons. ^c^θ: Watterson estimator of sequence diversity per site, π: average number of differences per site between two sequences, the diversity of the candidate genes was estimated for the non-coding (nc), synonymous (syn) and non-synonymous (nsyn) sites. ^d^Number of lineages with at least one singleton. ^e^D and F* were calculated for the simulation using parameters randomly drawn from the posterior distribution.^ f^ *P-*values were computed for unilateral tests as *P*< or > than the observed value according to the sign of the statistics without correction for multiple tests.

No differentiation between early and late landraces was detected at the three candidate genes (Table S4). It is noticeable that for *PgDwarf8*, the nucleotide diversity (π) of late landraces was twice the π value of early landraces (Table 4). On the contrary, for *PgPHYC*, the late landraces showed a lower level of nucleotide diversity (π) than the early landraces. No specific haplotypes fixed in either the early or in the late landraces have been identified in *PgHd3a*, *PgDwarf8 PgPHYC*. This was not surprising since the number of singletons was very high for all of these genes.

### Evidence for the Fingerprint of a Selective Sweep in Two of the Candidate Genes

It is noteworthy that tests for neutrality used in this study are conservative. This is a direct consequence for having simulating each gene using a broad range of values from the posterior distribution rather than the use of the point estimate values [86].

The results of the neutrality tests showed significant departures from selective neutrality in both *PgHd3a* and *PgDwarf8* in the domestic population but not in the wild population (Table 3 and Table 4). These two genes showed an excess of rare alleles compared to neutrality as witnessed by negative D_T_ and F* values. Because the distribution of D_T_ and F* values under the neutral hypothesis takes into account the inferred demographic history of the domestic population thanks to the modeling approach, we argue this result could be an evidence of a past selective sweep that has concerned these two genes in the domestic but not in the wild population. It was noticeable that *PgHd3a* showed a deviation from the neutral expectation in each domestic group (late and early landraces) (Table 3) whereas this was not the case for *PgDwarf8* since the neutrality tests were significant only in the late landraces (Table 4). This may suggest that *PgDwarf8* was targeted by selection only in the late group and not in the early group of landraces. This result could also be due to the lack of power to detect selection in the early landraces.

The polymorphism pattern in the *PgPHYC* fragment did not show any significant deviation from the neutral expectation. However, it is noticeable that the different subsamples exhibited contrasted results (Table 4). Indeed the early landraces and the wild populations showed negative values for D and F*, indicating therefore an excess of rare polymorphism. On the other hand the late domestic populations showed positive values of D and F* indicating an excess of allele at intermediate frequencies.

## Discussion

### The demographic History of Pearl Millet Domestication

Archeological data for pearl millet and other cereals allowed us to define a narrow window for the time of domestication. Indeed the beginning of domestication has been documented for wheat in the Fertile Crescent (10,500 to 9500 yr BP [87]), for rice in Asia (9000 to 7000 yr BP [88], [89]) and for maize in Meso-america (∼9000 yr BP [90]). Also for pearl millet, Manning *et al.* [58] have revealed that pearl millet could have been domesticated before 4500 years BP. These authors have also hypothesized that pearl millet cultivation could have begun 6000 years ago. It is also likely that wild grass harvesting, including *Pennisetum* species, was intensive 9000 years ago in the Sahel [59], [60]. These archeological dates were proved to be very useful to establish a calibration point for the estimation of the mutation rate in our model. This is witnessed by the high differences between the prior and the posterior distributions of µ. This estimation of a neutral mutation rate for pearl millet STS sequences will be very useful for future population genetics simulations.

Our molecular data showed that the domestic and wild pearl millet have similar amounts of nucleotide polymorphism. The domestic forms had seemingly lost only a small fraction (16%) of the neutral genetic diversity which is currently found in the wild populations of *Pennisetum glaucum*. This result is in accordance with those found in previous studies on pearl millet genetic diversity. Indeed, on the basis of microsatellite markers, Oumar *et al.* [12] showed that the domestic populations displayed 83% of the genetic diversity found in the wild populations, while it was 57% (estimation from θs values) in maize compared to teosinte [69]. In addition, Tajima’s D estimated on neutral STS loci were skewed towards negative values. Thus, domestic Pearl millet populations lack evidence of a bottleneck signature contrarily to what has been shown in sorghum [75] and in maize [69]. The demographic scenario inferred from our data was not fully conclusive in estimating the severity of the bottleneck. However, the upper bound of the credibility interval of the strength of the bottleneck estimation is hardly compatible with the level of genetic diversity we observed in the current domestic population relatively to the wild population. The recovery of genetic diversity in the domestic population could be partly explained by two evolutionary processes. First, the population expansion after the initial step of the domestication, could have given the opportunity for new mutations to accumulate in domestic populations. This expansion may contribute to explain the excess of rare alleles we observed on STS loci. However, this factor could have played only a minor role, taking into account the very short time that has elapsed since the beginning of domestication (only a few thousand of generations), and the mutation rate we estimated (6.5×10^−8^). Second, the migration of wild lineages into the domestic genetic pool may also explain the recovery of a significant amount of the genetic diversity after the initial bottleneck. The existence of gene flow between wild and domestic forms has been suggested by different studies [12], [80] and was supported by our simulation results. This gene flow may also explain why no fixed differences at the nucleotide level were found in either the wild or the domestic populations.

#### Genetic diversity within and among early and late landraces at neutral loci

This study revealed that the early and late landraces did not show significant genetic differentiation at the STS loci. The same level of genetic diversity was observed in both the early and the late landraces. These results could challenge the hypothesis of Tostain *et al.* [14] according to which the late landraces have evolved from a secondary diversification of early landraces at the west of the actual Lake Chad region. Other hypotheses on the origin of late landraces can be proposed. For example, they could have been the result of several independent local selection processes from early landraces all along the geographic distribution area of pearl millet. Yet, to our opinion, no strong arguments can actually be put forward against the fact that late landraces could have preceded early landraces. Indeed wild pearl millets are early flowering plants but nonetheless strongly sensitive to photoperiod as most wild plants in tropical areas. This characteristic ensures the coincidence between the rainy season and the life cycle of wild plants. Early landraces of pearl millet are also early flowering but much less photosensitive while late landraces have been shown to be late flowering and highly sensitive to day length [8], [10]. It is therefore difficult to single out a hypothesis about the evolutionary trajectories of both the cycle duration and the sensitivity to photoperiod, which have occurred within the cultivated gene pool since the beginning of pearl millet domestication.

The existence of a gene flow between early and late landraces could be another explanation for the absence of differentiation between these two phenological groups. Indeed gene flow between early and late pearl millets has been shown to occur in some areas in Niger by Lakis et *al.*, [11], and was also suggested by other authors [6], [91]. Lakis et *al.*, [11] have shown that this gene flow was driven by changes in farmers’ practices in response to social and environmental modifications. It is however not yet known whether this gene flow occurs all along their common geographic distribution area. Similarly, whether this gene flow is only a recent phenomenon driven by current changes or it has occurred for a long time in the past is also still to be elucidated.

We are conscious that the domestication scenario we simulated may be much simpler than the real domestication history of pearl millet. For example, multiple domestication events or a single domestication followed by a separation of early and late landraces may have occurred. In this study, we took into account the most common and simple hypothesis of a single domestication for pearl millet which has been proposed by several authors [12], [13], [92]. Yet, the evolutionary history of the wild population in our model is also likely oversimplified since we have hypothesized a constant wild population size since the domestication time. Major demographic events associated with climatic changes in the Sahelian region since the last millennium may have shaped the polymorphism pattern in the wild pearl millet populations. Thus, much more data and more knowledge on the history of the wild population are needed in order to simulate more complex scenarios of pearl millet domestication and to enhance the accuracy of parameter estimates. Nevertheless, the basic intent of our simulation work was to provide a more adequate model than the Wright-Fisher strict neutral model to test for selective sweeps in candidate genes.

### The Selection Regime for the Candidate Genes

Among the three candidate genes, two of them *PgHd3a* and *PgDwarf8* showed polymorphisms patterns that are compatible with a past event of directional selection in the domestic populations but not in the wild population. However we cannot exclude the possibility that the excess of rare alleles in comparison to the neutral expectation could be explained by the presence of a hidden genetic structure and unequal sampling in the domestic populations. Our analyses could have failed in detecting such a structure because of the moderate number of loci used in this study.

Another possibility is that recent gene flow from the wild population to the domestic genetic pool could have also affected the polymorphism frequency spectrum, by increasing the number of rare alleles, and consequently, the significant negative values for D_T_, and F* neutrality tests. In this case, clusters of singletons, corresponding to rare introgressed fragments, are expected to be found in a limited number of lineages. However, our data showed that singletons at both STS loci and candidate genes are distributed nearly equally among lineages (Table 1, Table 3 and Table 4). Thence, our data are in accordance with the occurrence of regular gene flow between wild and domestic populations since the beginning of domestication rather than rare introgression events.

An original output of our data is that it suggests that *PgHd3a* could have been the target of selection during the early phase of domestication before the phenological differentiation between early and late landraces. Indeed, *PgHd3a* showed a strong signal of a past selective sweep in both the early and the late landraces but not in the wild populations. Our results do not support a role of this gene, or at least of differences in the coding sequence, in the phenological differentiation between early and late pearl millets. This result confirms the results from Saïdou *et al.* [19] who have shown there was no correlation between polymorphisms in the coding sequence of *PgHd3a* and the variation in flowering time within a collection of pearl millet landraces from India, West and East Africa. However, in contrast to our study, they did not reject neutrality at this gene. The difference between the two studies could be a consequence of the very low level of nucleotide diversity [19] found at this gene (S = 7; θ = 0.16×10^−3^) in comparison with our data (S = 47; θ = 11.2×10^−3^), resulting in a lack of power to detect selection. However, the low polymorphism found by Saïdou *et al.* [19] could also itself be the consequence of a selective sweep. It is however noticeable that the level of nucleotide polymorphism was clearly lower for all the genes studied by Saïdou *et al.* [19] (0.16×10^−3^<θ<2.07×10^−3^) than for the three candidate genes in our study. Thus, this may also point at differences in sampling representativeness of the early and late pearl millet gene pools between the two studies.

Why could *PgHd3a* have been the target of selection in the whole domestic population but not in the wild? This gene plays a major role in the floral transition [36]. It has a well known function of integrating the different signals which promotes flowering [26] and several studies have demonstrated its implication in flowering time variation. For example, in wheat, Bonnin *et al.* [38] have revealed that orthologous of *Hd3a* could be involved in the variation of heading date within a collection of wheat inbred lines originated from various geographical origins. Also, in rice, Takahashi *et al.* [37] have shown that both mRNA expression level of *Hd3a* and molecular polymorphisms within its promoter region, are significantly associated with flowering time variation in a collection of cultivars. But more interestingly, these last authors have also shown that interaction effects between this gene and other flowering genes, *Ehd1* and *Hd1*, contributed largely to flowering time variation among rice cultivars. As stated above, the wild and the domestic pearl millet, display a different phenological habit. It is therefore possible that *PgHd3a* was involved in the phenological divergence between wild and domestic populations through selection that drove change in its function. Such a change could have been a prerequisite for phenological differentiation to occur later on within the domestic population through interaction between *PgHd3a* and other flowering genes. Further functional analyses of *PgHd3a* and studies of molecular polymorphisms in its promoter and in other genes within the same flowering gene network could provide more insights as to why *PgHd3a* was the target of selection in the domestic pearl millet as a whole.

In contrast with *PgHd3a, PgDwarf8* showed a significant signal of a past selective sweep only in the late landraces although this gene also showed negative D_T_ and F* values in the early landraces. This result could be due to a lack of power to detect selection in the early population. It could also suggest that *PgDwarf8* has been the target of human selection during a secondary step of the domesticated pearl millet evolution which could have been more specific to late landraces. Yet, it is noticeable that no specific alleles or haplotypes at *PgDwarf8* have been found in the late varietal group in comparison to early landraces and even to the wild pearl millets. Introgression of late landraces by alleles from the early domestic population could explain this last result. It is also possible that cis-regulatory sequences rather than the coding region were directly targeted by the selective event. Recombination between early and late haplotypes would therefore explain the absence of specific haplotypes in the coding region.

In maize, molecular polymorphisms in *Dwarf8* have already been shown to be associated with flowering time variation and to display the fingerprint of a selective sweep within a collection of 92 inbred lines [39]. It is noteworthy that Thornsberry *et al.* [39] included mainly “late” flowering populations of tropical and semi-tropical inbred lines in their study. The association of *Dwarf8* polymorphisms with flowering time variation was also confirmed in the early flowering elite European maize inbred lines [93]. These authors have also found a significant association between *Dwarf8* polymorphisms and plant height. This finding has led the authors to reconsider the role of *Dwarf8* in maize architecture. Indeed, mutants in orthologous of *Dwarf8*, in wheat (*Rht1*) [41], in barley (*Sln1*) [94], and in rice (*Slr1*) [95] have been identified as being able to decrease the responsiveness to Gibberellic Acid and accordingly to decrease plant height. It is therefore not possible to exclude the possibility that *PgDwarf8* may have been under selection within the domestic pearl millet gene pool because of its role in plant architecture rather than in flowering time variation.

Finally, the polymorphism within the studied region of *PgPHYC* (third intron) did not depart from strict neutrality in both the wild and the domestic populations. This suggests this gene was not involved in phenological evolution and diversification of pearl millet. However, in an experiment carried out by using a collection of 89 pearl millet lines, Saïdou *et al.* [19] have shown that molecular polymorphisms within the third intron of *PgPHYC*, was associated with variation in flowering time. They identified five haplotypes, among which three were found in very low frequency. The two other haplotypes have been found in intermediate frequencies leading to a deviation of the frequency spectrum from strict neutrality (Tajima’s D = 2,38 ; p<0,05). The authors suggested these two haplotypes could have been maintained by balancing selection targeting this gene through selection on cycle length. The pearl millet sample used in our study was much more genetically diverse at this gene. Indeed, 11 haplotypes were detected within the domestic populations among which the two major haplotypes found by Saïdou *et al.* [19]. These two haplotypes were also the most frequent in our sample of domestic landraces. As stated above, the difference between results obtained in these two studies could be due to differences in geographical representativeness of the two samples. Thus, it is possible, that *PgPHYC* has been the target of balancing selection only in some areas in Africa, or in some specific landraces. It was noticeable that, in the study of Saïdou *et al.* [19], the variation in flowering time was relatively low (overall mean = 58.8 days to female flowering (SE ± 0.54)). This range of variation is poorly representative of differences between early and late varieties according to farmers’ classification. Also, these two types of pearl millet are well known to differ in photoperiod sensitivity while differences among early landraces could rather mainly correspond to differences in intrinsic earliness [9]. Thence, differences between early and late landraces may not likely rely on differences of *PHYC* activity though this gene could contribute to variation in intrinsic earliness. Actually, the wider geographical area and the larger phenological variation covered by our sample could have hindered the detection of a selective sweep. Our data may also have revealed a weak fingerprint as a result of a composite sample including landraces that could have experimented different selection events impacting cycle evolution (i.e. different targeted genes or differences in time and/or intensity of the selective sweeps). Finally, in both studies, only the third intron of *PgPHYC* was studied. It is therefore not possible to exclude that other regions of this gene were targeted by human selection during the different steps of domestication.

## Supporting Information

Table S1 List of the primers used in this study for PCR amplification and sequencing. Legend: * Primers designed for this study. The same primers were used for PCR amplification and sequencing except internal primers for STS 738.
(PDF)

Table S2 List of accessions sequenced for the eight STS loci and the three candidate genes.
(PDF)

Table S3 Genetic diversity and tests for neutrality for the 8 STS loci. ^a^S: number of segregating sites. ^b^Sin: number of singletons. ^c^Specific sites: sites polymorphic in one population but monomorphic in the other. ^d^θ: Watterson estimator of sequence diversity per site, π: average number of differences per site between two sequences. ^e^Number of lineages with at least one singleton. ^f^D values were calculated using Fabsim, *P-*values of D were calculated for the standard coalescent model (*: p<0.05; ns: not significant). *P*-values were computed without correction for multiple tests.
(PDF)

Table S4 *F*st values between wild and domestic populations and between early and late landraces. *F*st values were estimated for each of the STS loci and the candidate genes. ^a^ *P*-values were computed without correction for multiple tests. (***:p<0.001; **: p<0.01; *: p<0.05; ns: not significant).
(PDF)

Table S5 Estimates of demographic and genetic population parameters obtained from the demographic model “without migration.” ^a^θ_W_ is the population mutation rate of the wild population, θ_D0_ is the population mutation rate of the domestic population at domestication time, θ_D1_ is the population mutation rate of the domestic population at present time, T is the time of domestication in units of 4Ne_W_ generations, and µ is the mutation rate per bp per generation. ^b^HPD interval is the interval of parameter values with the highest posterior density.
(PDF)
