# Supplementary material for: Evolution of Neutral and Flowering Genes along Pearl Millet (Pennisetum glaucum) Domestication
Source: PLoS One. 2012 May 14;7(5):e36642. doi: 10.1371/journal.pone.0036642 (PMC3351476; doi:10.1371/journal.pone.0036642)
Supplement: Table S1 — List of the primers used in this study for PCR amplification and sequencing. Legend: * Primers designed for this study. The same primers were used for PCR amplification and sequencing except internal primers for STS 738. (PDF) [file pone.0036642.s001.pdf]

Table S1. List of the primers used in this study for PCR amplification and sequencing.

Locus Name	Primer name	Sequence 5'-3'
**candidate genes primers**		
PgHd3a	FT F*	GGGACAGGGACCCGTTGG
	FT R*	GCGGGCCGAGGTGATAGAG
PgPHYC	phyc F*	ATCGTCTTCACCAGCTTCTGGCTT
	phyc R*	AACACACCAATCATCTTGTATTGGTC
PgDwarf8	F2d*	CTCGAGATGGCCATGGGGATGGG
	R2a*	GTGCGCTCAGTGCCCTCGCA
**STS loci primers**		
**STS 306**	STS 306 F	AACTTCCCAAATGAGAGATTGC
	STS 306 R	GAGAATCGCCTGCTGTGGTAG
**STS 344**	STS 344 F	CGTCCTGTACCTCAGCCTTG
	STS 344 R2*	CGTAATCTTCGATCCAACATCTGAT
**STS 359**	STS 359 F2*	GAGGTCGCCGACCTTGTATATATTC
	STS 359 R2*	GGCAGCCTAGGTCAATGAAAGG
**STS 476**	STS 476 F	GACCGAGCCTTCTTTCCTG
	STS 476 R	ACAAGTGCTGCTCAAAATTGG
**STS 521**	STS 521 F	ATTGGACAAGCAAATGAGCC
	STS 521 R	CAGCACCCAGCGTTTACTAC
**STS 713**	STS 713 F	CTAGTCCAAGCATTGTGAATC
	STS 713 R	TCTGGATGCACCATAACC
**STS 870**	STS 870 F	TGGAACATCTGAAGTGCCTCA
	STS 870 R	GGAGCTAAGCATAGAAGCAGCA
**STS 738**	STS 738 F	CATCAGCATGTGAGCATCG
	STS 738 R	TCCACAGTGAGCAACCTCAG
	Sequencing STS 738*	
	STS 738 intA F	TACTGAAAGGAGATCAACACGTTGC
	STS 738 intB R	CCTTACTCTCTGAACTTCTGCATC

* Primers designed for this study.
The same primers were used for PCR amplification and sequencing except internal primers for STS 738
