# Supplementary material for: Evolution of Neutral and Flowering Genes along Pearl Millet (Pennisetum glaucum) Domestication
Source: PLoS One. 2012 May 14;7(5):e36642. doi: 10.1371/journal.pone.0036642 (PMC3351476; doi:10.1371/journal.pone.0036642)
Supplement: Table S2 — List of accessions sequenced for the eight STS loci and the three candidate genes. (PDF) [file pone.0036642.s002.pdf]

Table S2. List of accessions sequenced for the eight STS loci and the three candidate genes.

Accession code	Type of population collected	Phenotype of the individual	Geographic origin	STS306	STS344	STS359	STS476	STS521	STS713	STS 738	STS870	*PgDwarf8*	*PgPHYC*	*PgHd3a*
115.98	Domesticated	Early	23DB	+	+	+	+	+	+			+		+
603.80	Domesticated	Early	Algeria				+							+
75.09	Domesticated	Early	Benin	+	+	+	+	+	+	+	+	+	+	+
310.90	Domesticated	Early	Benin				+		+					+
IRD2003-Tb5	Domesticated	Early	Benin	+		+	+	+	+		+	+		+
IRD2003-Tb6	Domesticated	Early	Benin					+						+
35.95	Domesticated	Early	Burkina-Faso	+	+		+	+					+	
53.09	Domesticated	Early	Burkina-Faso	+	+	+	+	+	+	+	+	+	+	+
133.98	Domesticated	Early	China	+	+	+	+	+	+		+			
604.80	Domesticated	Early	India				+				+			+
230.90	Domesticated	Early	India	+	+	+	+	+	+		+	+	+	
1390.84	Domesticated	Early	Kenya	+	+	+	+	+	+		+		+	+
223.90	Domesticated	Early	Mali	+	+	+	+		+					+
225.90	Domesticated	Early	Maroc											+
250.86	Domesticated	Early	Maroc	+		+	+	+	+	+	+		+	
IRD2003-Tb42	Domesticated	Early	Mauritania	+		+	+	+	+	+	+		+	+
72.88	Domesticated	Early	Mauritania				+				+	+		+
77.88	Domesticated	Early	Mauritania	+	+	+	+	+	+	+	+		+	
154.93	Domesticated	Early	Niger	+				+						
132.09	Domesticated	Early	Niger	+	+	+	+	+	+	+	+	+	+	+
156.09	Domesticated	Early	Niger	+	+	+	+	+	+	+	+	+	+	+
45.09	Domesticated	Early	Nigeria	+	+	+	+	+	+		+	+	+	+
492.76	Domesticated	Early	Nigeria									+		
459.76	Domesticated	Early	Nigeria	+	+	+	+	+	+	+	+		+	
175.79	Domesticated	Early	Ouganda		+			+	+	+			+	+
IRD2003-Tb67	Domesticated	Early	Pakistan	+	+	+	+		+				+	+
580.91	Domesticated	Early	Rajasthan				+							+
581.91	Domesticated	Early	Rajasthan	+							+			
206.98	Domesticated	Early	Senegal	+				+						
176.98	Domesticated	Early	Senegal	+	+	+	+	+	+	+	+	+		+
229.90	Domesticated	Early	Senegal	+	+	+	+		+		+			+
188.98	Domesticated	Early	Tchad	+		+	+	+	+		+	+		+
222.09	Domesticated	Early	Tchad	+	+	+	+	+	+		+	+	+	+
224.09	Domesticated	Early	Tchad	+	+	+	+	+	+		+	+	+	+
1427.84	Domesticated	Early	Tunisie		+	+	+		+	+	+		+	+
293.89	Domesticated	Early	Tunisie	+		+	+	+					+	+
78.09	Domesticated	Late	Benin								+		+	+
81.09	Domesticated	Late	Benin										+	
84.09	Domesticated	Late	Benin										+	
85.09	Domesticated	Late	Benin	+	+	+	+		+	+	+	+		+
86.09	Domesticated	Late	Benin	+	+	+	+	+	+	+				+
87.09	Domesticated	Late	Benin	+			+	+	+		+			
IRD2003-Tb7	Domesticated	Late	Benin		+		+							+
55.09	Domesticated	Late	Burkina-Faso	+	+	+	+	+	+	+	+	+	+	+
56.09	Domesticated	Late	Burkina-Faso	+	+	+	+	+	+	+	+	+	+	+
61.09	Domesticated	Late	Centrafrican Republic	+	+	+	+	+	+	+	+		+	+
62.09	Domesticated	Late	Centrafrican Republic	+	+	+	+	+	+		+	+		+
207.90	Domesticated	Late	Cameroun	+		+	+	+	+	+	+	+	+	+
208.90	Domesticated	Late	Cameroun				+							+
73.09	Domesticated	Late	Cameroun	+	+	+	+	+	+	+	+	+	+	+
74.09	Domesticated	Late	Cameroun								+			
25.95	Domesticated	Late	Côte d'Ivoire	+										
68.09	Domesticated	Late	Côte d'Ivoire	+	+	+	+	+	+		+	+	+	+
183.90	Domesticated	Late	Guinée				+							+
187.90	Domesticated	Late	Guinée				+		+					+
629.81	Domesticated	Late	Guinée											+
51.09	Domesticated	Late	Guinée								+		+	+
52.09	Domesticated	Late	Guinée	+	+	+	+	+	+	+	+	+		+
300.90	Domesticated	Late	Mali	+		+	+	+		+	+			
66.09	Domesticated	Late	Mali	+	+	+	+	+	+	+	+	+		+
65.09	Domesticated	Late	mali	+	+	+	+	+	+		+		+	+
65.88	Domesticated	Late	Mauritania		+		+		+	+				+
16.08	Domesticated	Late	Niger	+	+	+	+	+	+		+		+	+
77.08	Domesticated	Late	Niger	+	+	+	+	+	+		+			
48.09	Domesticated	Late	Nigeria	+	+	+	+		+		+	+	+	+
178.90	Domesticated	Late	Centrafrican Republic				+						+	
72	Domesticated	Late	Centrafrican Republic											+
69.09	Domesticated	Late	Senegal	+	+	+	+	+	+	+	+	+		+
70.09	Domesticated	Late	Senegal		+	+				+	+		+	
60.06	Domesticated	Late	Senegal									+		
61.06	Domesticated	Late	Senegal									+		+
63.06	Domesticated	Late	Senegal									+		+
44.09	Domesticated	Late	Tchad	+	+	+	+	+	+	+	+	+	+	+
211.9	Domesticated	Late	Togo			+								
67.09	Domesticated	Late	uganda	+	+	+	+	+	+		+	+	+	+
1.81	Domesticated	Undetrmined	Niger											
206.81	Domesticated	Undetrmined	Nigeria	+	+	+	+	+	+	+	+	+	+	
992.84	Domesticated	Undetrmined	Soudan	+		+	+	+	+		+			+
1057.84	Domesticated	Undetrmined	Tanzania				+		+					+
296.82	Domesticated	Undetrmined	Tanzania									+	+	
IRD2003-Tb88	Domesticated	Undetrmined	Tanzania	+	+	+	+	+	+		+	+		+
224.76	Domesticated	Undetrmined	Tchad	+		+	+	+	+	+			+	+
212.90	Domesticated	Undetrmined	Tchad						+	+				
169.81	Domesticated	Undetrmined	Zambia		+	+	+			+	+			+
IRD2003-Tb8	Wild		Benin									+		+
8.95	Wild		Burkina-Faso			+					+			
7.95	Wild		Burkina-Faso	+	+	+	+	+	+	+	+	+	+	
1717.84	Wild		Cameroun	+	+	+	+	+	+		+		+	+
IRD2003-Tb19	Wild		Cameroun									+		
9.95	Wild		Mali											+
289.92	Wild		Mali									+		
334.82	Wild		Mali	+	+	+	+	+	+		+		+	
10.95	Wild		Mali	+	+		+	+	+					
P8470	Wild		Mali	+	+		+	+	+	+	+	+	+	
P8473	Wild		Mali		+	+	+		+	+		+	+	+
P8475	Wild		Mali	+	+		+	+	+	+	+	+	+	
71.88	Wild		Mauritania	+		+		+	+		+			
60.88	Wild		Mauritania		+	+		+		+				
P8491	Wild		Mauritania			+			+	+	+		+	
P8493	Wild		Mauritania	+			+	+	+	+	+	+	+	
p8497	Wild		Mauritania		+	+	+	+	+	+	+	+	+	
2.95	Wild		Niger										+	
4.95	Wild		Niger	+	+	+	+	+	+	+	+	+		+
274 Alzou	Wild		Niger											+
41Alzou	Wild		Niger						+					+
307.90	Wild		Nigeria											
308.90	Wild		Nigeria	+		+	+	+	+	+	+			
327.95	Wild		Nigeria			+					+			
306.90	Wild		Nigeria	+		+		+	+		+		+	
IRD2003-Tb70	Wild		Centrafrican Republic	+	+	+	+	+	+	+	+	+	+	+
13.95	Wild		Senegal	+				+						
479.76	Wild		Senegal	+	+	+	+	+	+		+		+	+
357.82	Wild		Senegal	+				+	+	+	+	+	+	+
176.90	Wild		Soudan	+		+	+	+	+		+		+	+
177.90	Wild		Soudan			+			+		+			
214.90	Wild		Tchad											+
216.90	Wild		Tchad	+			+	+	+					+
16.95	Wild		Tchad			+			+		+			+
215.90	Wild		Tchad											
