# Supplementary material for: Evolution of Neutral and Flowering Genes along Pearl Millet (Pennisetum glaucum) Domestication
Source: PLoS One. 2012 May 14;7(5):e36642. doi: 10.1371/journal.pone.0036642 (PMC3351476; doi:10.1371/journal.pone.0036642)
Supplement: Table S3 — Genetic diversity and tests for neutrality for the 8 STS loci. aS: number of segregating sites. bSin: number of singletons. cSpecific sites: sites polymorphic in one population but monomorphic in the other. dθ: Watterson estimator of sequence diversity per site, π: average number of differences per site between two sequences. eNumber of lineages with at least one singleton. fD values were calculated using Fabsim, P-values of D were calculated for the standard coalescent model (*: p<0.05; ns: not significant). P-values were computed without correction for multiple tests. (PDF) [file pone.0036642.s003.pdf]

Table S3. Genetic diversity and tests for neutrality for the 8 STS loci.

		Sample size	Length	S[a]	Sin[b]	Specific sites[c]	π^d (10^{-3})	θ^d (10^{-3})	LSin[e]	Early/Late π ratio	Tajima's D[f]
STS 306	Early	26	563	5	2	2	2.55	2.33	1	1.93	0.27ns
	Late	21	563	3	0	0	1.32	1.48	0		-0.29ns
STS 344	Early	20	710	23	8	8	10.6	9.37	6	1.3	0.53ns
	Late	20	710	26	9	10	8.1	10.6	7		-0.89ns
STS 359	Early	23	440	11	2	3	7.18	7.34	2	0.97	-0.07ns
	Late	21	440	12	3	4	7.38	8.5	3		-0.12ns
STS 476	Early	29	752	6	3	1	1.96	2.04	2	0.77	-0.11ns
	Late	26	752	9	4	2	2.53	3.15	4		-0.63ns
STS 521	Early	24	617	23	16	12	7.1	10.69	9	1.48	-1.23ns
	Late	18	617	14	10	5	4.81	7.05	3		-1.19ns
STS 713	Early	24	1065	17	13	13	2.18	4.45	8	0.62	-1.83*
	Late	21	1065	16	5	11	3.49	4.24	3		-0.65ns
STS 738	Early	11	1995	31	16	15	4.7	5.35	7	0.92	-0.56ns
	Late	14	1995	50	35	33	5.1	8.01	14		-1.59ns
STS 870	Early	22	744	23	10	8	8.16	9.42	6	1.04	-0.50ns
	Late	23	744	25	14	9	7.8	10.09	7		-0.85ns
Average	Early	22	860	17	9	8	5.55	6.37	5	1.13	-0.44
	Late	20	860	19	10	9	5.1	6.64	5		-0.78

[a]S: number of segregating sites. [b]Sin:number of singletons. [c]Specific sites: sites polymorphic in one population but monomorphic in the other. [d]θ: Watterson estimator of sequence diversity per site, π: average number of differences per site between two sequences. [e]Number of lineages with at least one singleton. [f]D values were calculated using Fabsim, *P*-values of D were calculated for the standard coalescent model (*: $p<0.05$; ns: not significant). *P*-values were computed without correction for multiple tests.
