# Supplementary material for: Evolution of Neutral and Flowering Genes along Pearl Millet (Pennisetum glaucum) Domestication
Source: PLoS One. 2012 May 14;7(5):e36642. doi: 10.1371/journal.pone.0036642 (PMC3351476; doi:10.1371/journal.pone.0036642)
Supplement: Table S4 — Fst values between wild and domestic populations and between early and late landraces. Fst values were estimated for each of the STS loci and the candidate genes. a P-values were computed without correction for multiple tests. (***:p<0.001; **: p<0.01; *: p<0.05; ns: not significant). (PDF) [file pone.0036642.s004.pdf]

Table S4. *F*st values between wild and domestic populations and between early and late landraces.

	Wild vs Domestic[a]	Early vs Late[a]
STS 306	0.288***	0.177**
STS 344	0.26***	0.10*
STS 359	0.09**	0.035ns
STS 476	0.11***	-0.013ns
STS 521	0.021ns	0.029ns
STS 713	0.081**	0.03ns
STS 738	-0.016ns	-0.03ns
STS 870	0.127***	0.009ns
Average	0.12	0.04
PgHd3a	0.12***	-0.008ns
PgDwarf8	0.095*	-0.026ns
PgPHYC	0.077ns	0.042ns

*F*st values were estimated for each of the STS loci and the candidate genes. [a]*P*-values were computed without correction for multiple tests. (***:p<0.001; **: p<0.01; *: p<0.05 ; ns: not significant).
