# Supplementary material for: Evolution of Neutral and Flowering Genes along Pearl Millet (Pennisetum glaucum) Domestication
Source: PLoS One. 2012 May 14;7(5):e36642. doi: 10.1371/journal.pone.0036642 (PMC3351476; doi:10.1371/journal.pone.0036642)
Supplement: Table S5 — Estimates of demographic and genetic population parameters obtained from the demographic model “without migration.” aθW is the population mutation rate of the wild population, θD0 is the population mutation rate of the domestic population at domestication time, θD1 is the population mutation rate of the domestic population at present time, T is the time of domestication in units of 4NeW generations, and µ is the mutation rate per bp per generation. bHPD interval is the interval of parameter values with the highest posterior density. (PDF) [file pone.0036642.s005.pdf]

Table S5. Estimates of demographic and genetic population parameters obtained from the demographic model “without migration”.

Parameter[a]	median	95%HPD interval[b]
θ_W	0.0061	0.0036 – 0.0106
θ_{D0}	3.91×10^{-4}	3.05×10^{-5} – 0.0042
θ_{D1}	0.1015	0.0080 – 3.4419
T	0.0295	0.0082 – 0.0809
μ	2.52×10^{-8}	7.98×10^{-9} – 7.66×10^{-8}

[a] θ_W is the population mutation rate of the wild population, θ_{D0} is the population mutation rate of the domestic population at domestication time, θ_{D1} is the population mutation rate of the domestic population at present time, T is the time of domestication in units of $4Ne_W$ generations , and μ is the mutation rate per bp per generation. [b] HPD interval is the interval of parameter values with the highest posterior density.
